# Critical evaluation of colon submucosal microdialysis in awake, mobile rats

**DOI:** 10.1371/journal.pone.0191041

**Published:** 2018-01-11

**Authors:** Norbert Cibicek, Jiri Ehrmann, Jitka Proskova, Rostislav Vecera

**Affiliations:** 1 Department of Medical Chemistry and Biochemistry, Faculty of Medicine and Dentistry, Palacky University, Olomouc, Czech Republic; 2 Department of Histology and Embryology, Faculty of Medicine and Dentistry, Palacky University, Olomouc, Czech Republic; 3 Department of Clinical Biochemistry, University Hospital, Olomouc, Czech Republic; 4 Department of Pharmacology, Faculty of Medicine and Dentistry, Palacky University, Olomouc, Czech Republic; National Health Research Institutes, TAIWAN

## Abstract

Sensors able to record large bowel physiology and biochemistry *in situ* in awake rodents are lacking. Microdialysis is a mini-invasive technique that may be utilized to continuously deliver or recover low-molecular substances from various tissues. In this experiment we evaluated the feasibility of *in vivo* microdialysis to monitor extracellular fluid chemistry in the descending colon submucosa of conscious, freely moving rodents. Following surgical implantation of a microdialysis probe, male Wistar rats were housed in metabolic cages where they were analgized and clinically followed for four days with free access to standard diet and water. To assess local microcirculation and probe function, glucose, lactate, glucose-to-lactate ratio and urea clearance were determined in the dialysates from the three postoperative days with focus on the final 24-h period. In an attempt to mitigate the expected tissue inflammatory response, one group of animals had the catheters perfused with 5-aminosalicylic acid-enriched medium with final concentration 1 μmol/L. For verification of probe position and the assessment of the surrounding foreign body reaction, standard histological and immunohistochemical methods were employed. Microdialysis of rat gut is associated with considerable technical challenges that may lead to the loss of probe function and high drop-out rate. In this setting, limited data did not allow to draw any firm conclusion regarding local anti-inflammatory effectiveness of 5-aminosalicylic acid perfusion. Although intestinal microdialysis may be suitable for larger anesthetized animals, low reproducibility of the presented method compromises its routine experimental use in awake and freely moving small-sized rodents.

## Introduction

Intestinal metabolism, barrier function and blood perfusion are interrelated and relevant for understanding of the relationship between nutrition, medication, health and disease [1–5]. Owing to gut functional anatomy, the methods to record biochemistry, examine physiology or continuously track drug levels on the local level have been limited. Experiments were restricted to *ex vivo* preparations [6,7] or to *in vivo* models performed under general anesthesia [1,8–12]. Some human studies were performed in the post-operative recovery phase [13,14]. Research involving awake small rodents has been focused on stomach or peritoneal cavity with up to several-hour monitoring [15–17]. To the best of our knowledge, no attempts to monitor the (large) bowel *per se* in freely moving rats have been hitherto published.

Microdialysis is a versatile miniinvasive sampling technique utilized in numerous experimental and clinical settings [8,13,14,18]. The sensing is vitally dependent on a special probe (or catheter) containing semipermeable membrane and implanted in a tissue or fluid of interest where, at least in principle, it mimics a blood capillary. Advantage of this approach lies in the possibility to examine extracellular fluid chemistry continuously for number of hours or days *in situ* without necessity to either sacrifice the animal during sample harvesting or to withdraw any cellular matter. Such practice might lead, besides high resolution metabolic or pharmacokinetic profiles, also to a reduction of the number of experimental subjects. Based on simple diffusion, the catheter allows continuous delivery or withdrawal of metabolites [19,20], molecules related to cellular damage [2], markers of intestinal permeability [2,3], blood flow indicators [20,21], signaling molecules [15,22], medications [23], or different (tracer-labelled) internal standards used for calibration purposes [24]. Thus, if successfully applied in the gut of awake rodents, microdialysis-based sensors might prove advantageous for various priorities in experimental animal research.

The aim of the present study was to modify a previously published method of colon submucosal microdialysis carried out under general anesthesia with a concentric probe type [3] and investigate the performance and applicability of a novel protocol based on a thinner, more flexible and thus less injurious linear microdialysis catheter in conscious, mobile rats. We hypothesized that the implantation of the probe would allow for reproducible measurements of local blood perfusion and tissue biochemistry using glucose, lactate, glucose-to-lactate ratio and urea clearance. In effort to minimize the expected tissue inflammatory response, 5-aminosalicylic acid (5-ASA) was added into the perfusion medium.

## Materials and methods

### Materials

As microdialysis perfusate A argon-deoxygenated (5 min) and urea-enriched (final concentration 20 mmol/L) phosphate-buffered saline (PBS, pH 7.4) was used. 5-ASA, also known as mesalazine (Sigma-Aldrich), was added to this solution (final concentration 1 mmol/L) as an anti-inflammatory substance to produce microdialysis perfusate B. Both solutions were prepared on day 1 and used throughout the whole experiment. Five-day stability of these solutions without precipitate formation was verified spectrophotometrically at room temperature and exposure to daylight.

### Animals

In the present study, altogether 20 adult SPF male rats of Wistar strain (*Rattus norvegicus*, 339–387 g, 11–14 weeks old), originally from Envigo (formerly Harlan), Italy (purchased from Anlab, ltd., Prague, Czech Republic, at delivery weight of 275–349 g, 9–12 weeks old), were used. During acclimatization period (at least 7 days before experiment), the animals were housed in animal quarters (Center for Work with Laboratory Animals of the Palacky University, Medical Faculty in Olomouc, Czech Republic) in standard plastic cages with dust-free bedding (sawdust), 2 rats per cage, under controlled environmental conditions with free access to tap water and standard rat chow (pellets from Ssniff^®^ R/M-H, Ssniff Spezialdiäten GmbH, Soest, Germany).

### Ethical issues

All protocols and experimental procedures of the study were approved by the authors' Institutional Animal Care and Use Committee and the Ethics Committee of the Ministry of Education, Czech Republic (Approval Number: 11732/2014-5). All animals received humane care in compliance with the Experimental Animals Protection Act No. 167/1993 L.C. Surgery was performed under general anesthesia and all efforts were made to minimize suffering, including the use of analgesics and euthanasia. The number of animals was a minimum enabling, in case of success, a meaningful statistical assessment. If technical failure occurred, the particular experiment was discontinued to avoid futile, unethical examinations.

### Experimental setup

The experiment lasted for five days. Prior to randomization, the animals were fasted for 18 to 24 h with free access to tap water. On day 1 they were weighed using an electronic balance (DL-3, Denver Instrument, Denver, Colorado, USA) and randomly assigned to two experimental groups—group A (*n* = 10), group B (*n* = 10). Following randomization, each rat from groups A and B was implanted a microdialysis catheter and was introduced into its own metabolic cage (Tecniplast, Buguggiate, VA, Italy). The experimental groups differed in the quality of perfusion medium used for microdialysis–group A was given perfusate A and group B obtained perfusate B (containing 5-ASA). All subjects were monitored regarding water and food intake, stools and urine output and stools water content until the end on day 5.

### Surgery

After weighing, the animals were intramuscularly injected with a mixture of ketamine (Narkamon^®^, Spofa, Prague, Czech Republic, 80 mg/kg_b.wt._) and xylazine (Rometar^®^, Spofa, Prague, Czech Republic, 10 mg/kg_b.wt._). An intraperitoneal bolus of midazolam (Dormicum^®^, Roche ltd., Prague, Czech Republic, 1 mg/kg_b.wt._) and a subcutaneous prophylactic dose of antibiotics (enrofloxacine, Enroxil^®^, Krka, Novo Mesto, Slovenia, 10 mg/kg_b.wt._) were administered. Thereafter the eyes were covered with unguent (guaiazulene, Ophthalmo-Azulen^®^, Zentiva, Prague, Czech Republic) to avoid corneal dessication. The animals were placed onto a hotplate (HT 01, Harry Gestigkeit GmbH, Düsseldorf, Germany) adjusted to 38°C to prevent heat loss. The abdomen and nuchal region were disinfected with iodine-based solution.

Firstly, following midline incision of the skin at the nuchal region and the middle abdomen, a subcutaneous tunnel was made with forceps to connect both regions and enable the passage of a plastic sleeve. The sleeve was used to guide, situate and aid the fixation of the Dacron^®^ mesh button (Instech Solomon, Plymouth Meeting, PA, USA) between the animal´s ears for subsequent sutures. Secondly, the descending colon was identified and stretched using a needle-holder to assist the ensuing procedures. Ca. 7 cm from the rectum, a 10–12 mm submucosal tunnel was formed using 27G needle from the serosal side with caution not to disrupt the serosa nor mucosa. The needle was then used to guide the probe´s fiber skeleton. Thereafter, the microdialysis probe LM-5 (5 mm, 30 kDa MW cut-off polyacrylonitrile membrane, BaSi, West Lafayette, IN, USA) was drawn through the tunnel finally extracting the needle. The probe was fixed in place using two atraumatic sutures (silk braided black, DR8 needle, USP 7/0, Chirmax ltd., Prague, Czech Republic)–one with fully stretched gut, the other followed after the needle-holder was partially tightened making the gut more relaxed. Thirdly, the colon was relocated *in situ* and the animals were rehydrated with intraperitoneal bolus of sterile crystalloid (Plasmalyte^®^, Baxter Czech ltd., Prague, Czech Republic, 10 mL/kg_b.wt._ at 37°C). The probe´s tubing was glued with an adaptor tube by two-component epoxy adhesive (UHU plus 2 min sofortfest epoxy, UHU GmbH & Co., Bühl, Baden, Germany) to allow its subsequent connection to standard microdialysis FEP tubing using silicone tubing connectors (both BaSi, West Lafayette, IN, USA). Thereafter the tubing was pulled through the subcutaneous tunnel and the abdomen was sutured in two layers. Fourthly, the catheter and the FEP tubing were connected outside the animals and tubing patency was tested with sterile water. Finally, the external tubes were secured using spring tether (BaSi, West Lafayette, IN, USA) and the skin was disinfected with iodine. Total duration of surgery was ca. 50 min (S1 and S2 Figs).

In the post-operative period, the animals were subcutaneously injected with analgesic (ketoprofen, Ketofen^®^, Merial, Toulouse Cedex, France, 5 mg/kg_b.wt._) and placed in the respective metabolic cages onto aluminum foil to reduce heat loss. During the recovery phase, the rats´ temperature was constantly monitored and maintained at 37 to 38°C using automatic, rectal probe-controlled heating lamp (TCAT-2AC controller, Physitemp instruments Inc., Besozzo, VA, Italy).

### Microdialysis

**Fig 1:**
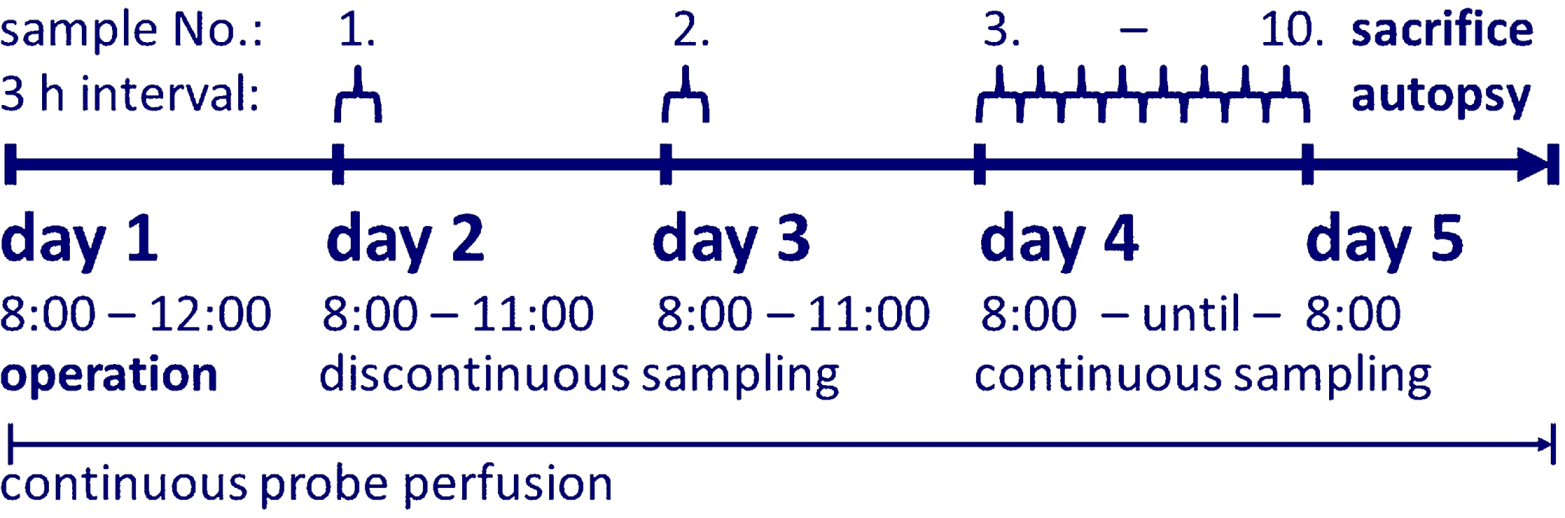
Experimental design. Sampling pattern reflected our interest in the fourth 24-h period (day 4). Information from preceding time periods was intentionally restricted to two solitary measurements.

As soon as the animals regained consciousness the body heating was ceased and the microdialysis system was connected with the infusion pump (PHD 2000, Harvard apparatus, Holliston, MA, USA) via a dual channel swivel mounted on a counter balance arm (both from Instech Solomon, Plymouth Meeting, PA, USA, S3 Fig). Continuous perfusion flow-rate was set at 1 μL/min, the solution was maintained at room temperature and the dialysate was collected into sterile Eppendorf tubes in 3 h intervals as shown in Fig 1. The collected material was stored at -80°C until analysis of glucose, lactate and urea on a clinical chemistry platform (Aution Max analyzer, Arkray, Japan). Glucose and lactate were used to calculate lactate-to-glucose ratio as reported previously [20]. At the cessation of experiment, relative delivery of urea (RD_urea_, expressed in %) was computed as follows:

$$RD(urea)=\frac{c(urea)dialysate‑c(urea)perfusate}{c(urea)ECF‑c(urea)perfusate}\;100(\%)$$ (1)

Where c(urea)_dialysate_ represents the concentration of urea in the microdialysate, c(urea)_perfusate_ stands for the concentration of urea in the perfusate and c(urea)_ECF_ symbolizes the concentration of urea in the extracellular fluid surrounding the catheter. Due to supposedly equal distribution of unbound urea in the extracellular space, we assumed the latter to be insignificantly different from the concentration of urea determined in the blood plasma [25]. RD_urea_ (%) was expressed as median (min-max) of values obtained on day 4.

Functionality of the microdialysis probe type used for *in vivo* experiments was tested *in vitro* (at 25°C) for relative recovery of urea in a set of no-net-flux calibration experiments according to Lönnroth [26]. As perfusates, urea-enriched Ringer´s solutions (containing 0, 3, 5, 8, 11, 15 and 19 mmol/L urea, Sigma-Aldrich, USA) were used. Seven LM-5 probes were immersed in 100 mL of medium containing Ringer´s solution with 10 mmol/L urea. Catheters were perfused at 0.2, 0.5, 1, 2 and 5 μL/min (S5 Fig).

### Clinical examination

In the postoperative period, all animals had free access to standard rat pellets and daily fresh tap water. Physiological parameters, *i*.*e*. 24-h feed consumption (g/kg_b.wt._), water intake (mL/kg_b.wt._), urine (mL/kg_b.wt._) and stools (g/kg_b.wt._) output, stools quality and water content (%) were documented for both groups (from day 2 to day 5). Animal health, body condition and well-being were assessed by experienced physicians (NC, RV) or a veterinary doctor (DD) twice daily (in the morning and in the afternoon) based on a combination of clinical signs (wakefulness, breathing, behavior such as active posture and self-care, fur quality, wound healing) and physiological parameters.

Provided painful reaction or dehydration the animals were subcutaneously analgized by a non-steroidal anti-inflammatory drug (Ketofen^®^, 5 mg/kg_b.wt._) or rehydrated by the same route using balanced crystalloid solution (Plasmalyte^®^, 10 mL/kg_b.wt._). On condition that this treatment would not bring recovery within 12–24 h, or if the animal would present with lethargy, anorexia or signs of fresh bleeding, wound dehiscence or suppuration or in the case of microdialysis setup failure, the animal would be anesthetized by intramuscular injection of ketamine (Narkamon^®^, 80 mg/kg_b.wt._) and xylazine (Rometar^®^, 10 mg/kg_b.wt._) with subsequent euthanasia by cardiac intraventricular injection of T61^®^ solution (Intervet International GmbH, Unterschleissheim, Germany, 3 mL/kg_b.wt._) carried out by a veterinary doctor (DD).

On day 5, subsequent to routine material and data collection, all animals were weighed, intramuscularly anesthetized with a mixture of fentanyl (40 μg/kg_b.wt._) and medetomidin (200 μg/kg_b.wt._) followed by diazepam (5 mg/kg_b.wt._). Animals were sacrificed by withdrawal of arterial blood from the aortal bifurcation.

### Stools water content

Each morning from day 2 to day 5, all stools were removed from the cage, packed in airtight plastic sachets and analyzed for water content on the day of collection using Ohaus MB25 moisture balance (Ohaus Corp., Parsippany, USA).

### Biochemistry and hematology

Arterial blood was collected into K_2_EDTA tubes and divided into two aliquots. Following blood centrifugation of the first aliquot (10 min at 450 × g and 4°C), plasma was isolated and immediately stored at -80°C. Glucose, lactate and urea concentrations were determined within one month on a routine clinical chemistry platform (Aution Max analyzer, Arkray, Japan) in compliance with current national standards for quality requirements. The second aliquot was utilized, without delay, for basic blood count examination applying Vet ABC Hematology Analyzer (Horiba ABX, France) in accordance with the manufacturer´s instructions.

### Histology

The abdominal cavity, the viscera and the catheter were thoroughly inspected (S4 Fig). After isolation, longitudinal dissection and washing in ice-cold phosphate-buffered saline (pH 7.4), colon was measured as to its length, photographed and sampled for probe position verification and assessment of foreign body reaction. The detached materials were fixed in 10% formaldehyde and treated according to standard protocols for hematoxylin-eosin and Alcian blue staining. Moreover, inducible Nitric Oxide Synthase (iNOS) immunoreactivity was determined immunohistochemically. Briefly, the iNOS protein was detected in an indirect two-step assay with rabbit polyclonal antibodies (anti-NOS-2 antibody, sc-8310; Santa Cruz Biotechnology, Dallas, TX, USA). The antibody was diluted 1:100 in Dako REALTM Antibody Diluent (Dako). Following slide deparaffinization and hydration, heat antigen retrieval was performed to unmask the antigen. Then, all slides were incubated with primary antibody for 1 h at room temperature. The ensuing detection was performed by EnVisionTM Detection System, Peroxidase/DAB, Rabbit/Mouse (Dako). Tris-HCl buffer (pH 7.6) was used for washing between the steps. Nuclei were counterstained with hematoxylin. After dehydration, the samples were cover-slipped, coded to minimize observer bias and semi-quantitatively evaluated under light microscope. Each slide was assessed by a professional pathologist (JE) blinded to the study protocol and scored on a negative (–, no detected signal), mild (+), moderate (++) to strongly positive (+++) signal scale.

### Statistics

Statistical analyses were performed using GraphPad InStat software version 3.06 (GraphPad Software, San Diego, CA, USA). Unless otherwise noted, data are, due to low number of subjects, presented as single measurements. Therefore, subjects are considered on individual basis. Concerning respective parameters monitored by microdialysis, statistical approach was restricted to nonparametric methods. Differences between animals were assessed by Kruskal-Wallis test followed, where applicable, by Dunn´s *post hoc* tests. Spearman´s correlation coefficient was calculated in order to determine possible relationships between microdialysis parameters with focus on glucose or lactate-to-glucose ratio *vs*. urea or RD_urea_. In each case, two-tailed p-value was considered. Selected level of statistical significance was p<0.05.

## Results

### Awake animal model of colon submucosal microdialysis

As shown in Table 1, the introduction of the model was associated with high drop-out rate. One animal died spontaneously shortly after operation (day 1), still under general anesthesia. Two rats had to be euthanized on the following day–one because of lethargy and another due to dehiscence of laparotomy. Another two animals had their probes re-implanted immediately after the first implantation due to unacceptable extent of hemorrhage developing within the original submucosal tunnel. Besides complications resulting from the operation and/or anesthesia, on most occasions, failure of the technique was attributable to the sensor (layout), its dislocation or its constituents *per se*. Technical problems with the flow of dialysate were in one incident combined with the subject´s demise as the swivel entangled itself in microdialysis tubing. Since this accident developed overnight (between days 2 and 3), the affected individual could not be euthanized. From the remaining 16 animals only ten had normal dialysate flow (so another six subjects with altered microdialysis fluidics had to be excluded from the study and euthanized). Catheter dislodgement was predominantly intraluminal and occurred in four out of six animals with discontinued flow of microdialysate and in three out of ten normally perfused probes. Therefore, normal dialysate flow did not systematically indicate correct probe position and, by analogy, insufficient catheter perfusion or flow cessation was not always a result of sensor displacement with subsequent perfusate contamination and catheter obstruction. Owing to (autopsy-verified) probe dislocation the three subjects with normal dialysate flow had to excluded from further analyses. Implantation of the microdialysis sensor was almost regularly accompanied by altered bowel physiology. Although considered significant, certain clinical findings (such as soft stools, lower spontaneous fluid intake or slow bowel movements) that did not lead to dehydration, macroscopic bleeding or severe clinical alteration with obvious pain perception were not imperative for euthanasia. Post-mortem examination revealed that even strictly submucosal probe position was often (in six out of seven rats) associated with more or less significant pathology leading to further drop-outs from the study. The impairments included sticky stools (one rat), serious degree of constipation (invalidating three individuals), subcutaneous seroma (one animal), localized abscess (excluding one subject) and aortal aneurysm (one rat that was already disqualified due to coprolit). Since soft stools and small localized seroma did not have marked impact on the animals´ physiology, the two rats were also considered eligible for complex data evaluation. Thus, the final number of animals remaining in the study (and presented further on in this paper) was three.

**Table 1:** Summary of experimental challenges.

Reason of failure • No. of remaining animals (time of demise)	No. of animals with given failure	Relative No. of animals with given failure
**Complication during operation or due to anesthesia** • leading to exit during anesthesia in 1 rat (day 1), and euthanasia in 2 rats (day 2) • 17 (= 20–3) animals survived operation and anesthesia	5	5 out of 20 enrolled and operated
**Failure of microdialysis tubing or flow of dialysate** • leading to spontaneous death in 1 rat (between days 2 and 3) • 10 (= 17–7) animals continued with normal dialysate flow • 6 (= 16–10) rats were euthanized for abnormal medium flow	7	7 out of 17 survivors
**Probe dislocation verified at autopsy** • altogether 5 luminal and 2 peritoneal dislocations • 7 (= 10–3) rats remained with normal dialysate flow and catheter position	7^a^	3 out of 10 with normal dialysate flow 4 out of 6 with abnormal dialysate flow
**Other significant post-mortem findings**^**b**^ • 1^c^ rat had normal dialysate flow, probe position and no significant tissue or stools quality alteration	12^a^	6 out of 7 with normal medium flow and correct sensor position 6 out of 9 with abnormal dialysate flow or dislodged catheter

^a^Autopsy was not performed in four animals that died or were euthanized in the first two to three days of experiment.

^b^Other significant findings included soft stools, ileus or stercolith, seroma, arterial aneurysm or intraperitoneal abscess.

^c^Soft stools or mild subcutaneous seroma, albeit classified as significant findings, were not considered clinically severe. They were present in two animals out of seven with normal dialysate flow and catheter position, so finally three animals remained in the experiment instead of one.

### Microdialysis data

**Fig 2:**
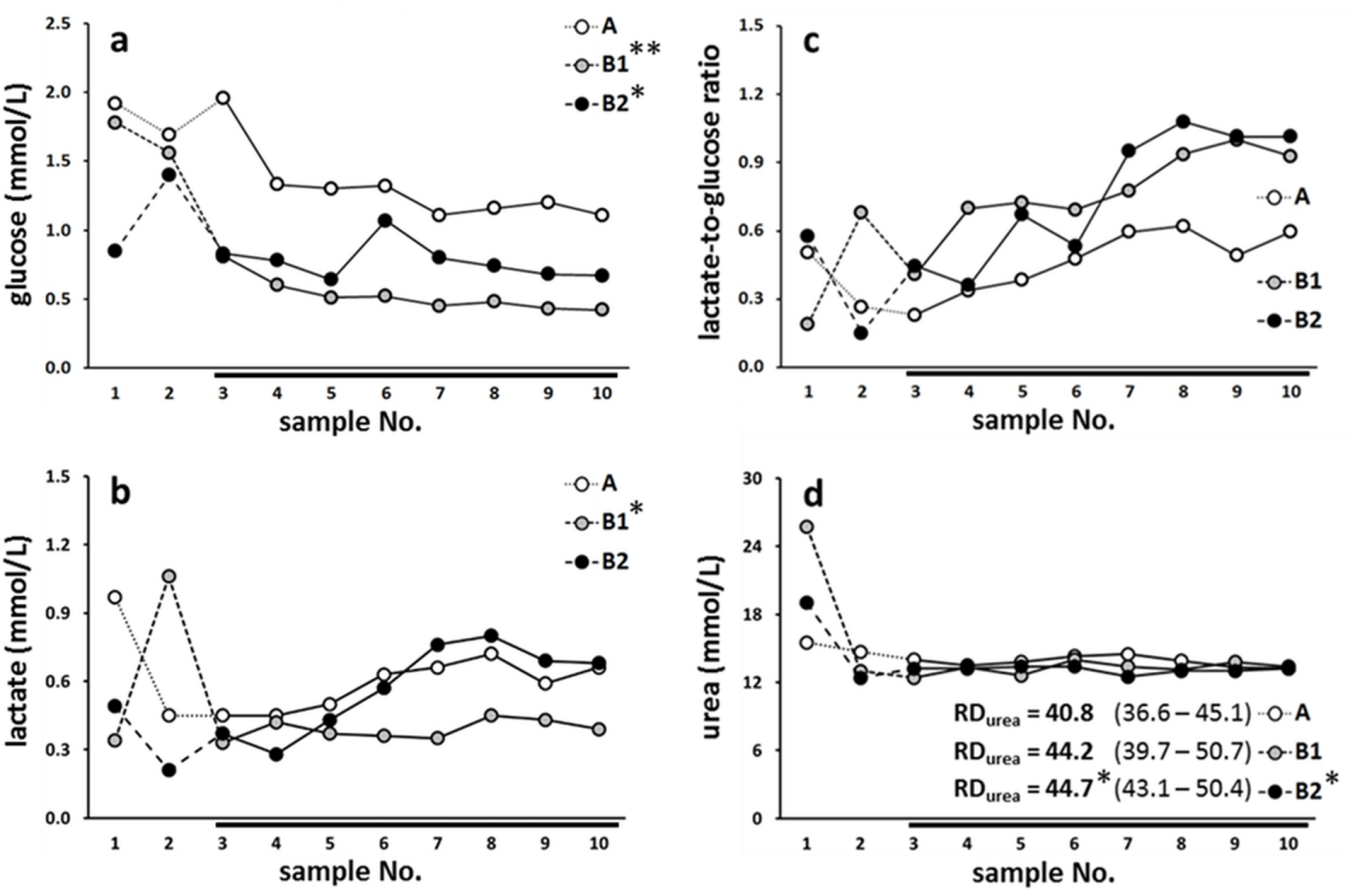
Microdialysis data. Panels a-d depict data from three eligible animals (one from group A with perfusate A and two from group B with anti-inflammatory perfusate B). Data are displayed as individual measurements per time interval. RD_urea_ (%) was expressed as median (min-max) of values obtained on day 4. Symbols denote: *p<0.05 and **p<0.01 *vs*. rat A. Statistical approach was restricted to the evaluation of inter-individual differences owing to low number of subjects. Possible effect of 5-ASA (perfusate B) could not be determined. Due to ethical reasons the attempts to harvest more data were discontinued.

Data from the three eligible animals—one from group A (perfusate A) and two from group B (anti-inflammatory perfusate B) with normal microdialysate flow, correct probe position and absence of ileus or other severe complications are presented in Fig 2. In all three subjects (A, B1 and B2), progressive decrease in dialysate glucose concentration was accompanied by a slow, yet steady increase in lactate-to-glucose ratio and almost constant levels of urea. For glucose, lactate and urea, significant differences were found between within-subject *vs*. between-subject data variations (as for lactate-to-glucose ratio, p-value equaled 0.055). Rat A had significantly higher microdialysate glucose than rats B1 and B2. Rat A also had significantly higher lactate than rat B1, although lactate-to-glucose ratio of rat A was the lowest from all three animals. Urea in rat A was significantly higher and RD_urea_ (calculated from values obtained on day 4) was significantly lower than the respective values in rat B2, albeit the differences were marginally significant. Regarding microdialysis parameter correlations, we focused our attention to glucose or lactate-to-glucose ratio *vs*. urea or RD_urea_. In the eligible animals no expressive relationships could be described between these markers (Table 2).

**Table 2:** Correlations between microdialysis parameters.

Correlation between parameters	Urea	RD_urea_
	r value (95% CI)	r value (95% CI)
• Glucose	0.352 (-0.021 to 0.639)	-0.306 (-0.639 to 0.124)
• Lactate-to-glucose ratio	-0.337 (-0.629 to 0.038)	0.354 (-0.070 to 0.670)

In the eligible animals (A, B1 and B2), no significant correlations between glucose, lactate-to-glucose ratio, urea and RD_urea_ could be observed (p>0.05). Abbreviations: CI–confidence interval, RDurea−relative delivery of urea.

### Animal physiology and body weight

The impact of the experimental technique on animals´ water intake, urine output, feed consumption, stools wet weight, stools water content and body weight is summarized in Table 3. The operation generated slightly negative and comparable responses in terms of weight gain (change from 0 to—5%). The dynamics in water or feed intake and urine or stools output was globally positive or stabilized. However, considerable intra- and inter-individual variations were encountered, with animal A presenting with the least post-surgical recovery. Delayed beginning of water intake and feed consumption could be noticed also in subject B1, however, with a clearer trend towards compensation on the following days. Anuria was not present in any rat. In all subjects stools consistency varied from soft formed excrements to hard feces. Although stools water content largely reflected the amount of released material rather than the actual damp, water content did not exceed 60% even in the bulkiest stools indicating the absence of diarrhea. The stools contained no visible blood.

**Table 3:** Animal physiology.

Day	1	2	3	4	1	2	3	4	1	2	3	4	0	4
Parameter (unit)	Water intake (mL/kg_b.wt._/24h) Urine output (mL/kg_b.wt._/24h)				Feed consumption (g/kg_b.wt._/24h) Stools wet weight (g/kg_b.wt._/24h)				Stools water content (%)				Abs. body wt. (g) Rel. body wt. (%)	
**A**	0.0 22.7	102 67.0	81.0 37.8	58.5 23.4	6.2 0.0	12.1 11.5	15.9 5.7	29.1 6.5	-	44.7	46.5	47.9	382 100	364 95
**B1**	0.0 14.6	194 106	91.1 60.1	109 49.0	5.8 0.0	17.8 16.9	32.7 19.0	30.2 9.8	-	57.5	57.0	45.1	361 100	352 98
**B2**	101 15.4	112 36.3	57.0 25.6	77.7 36.3	10.5 1.6	24.8 15.1	33.6 16.0	41.7 13.5	37.7	47.7	60.0	49.1	359 100	358 100

Due to low number of subjects, statistical evaluation was not performed, and data were expressed as individual measurements. In compliance with ethical principles, experiments attempting to harvest more data were not continued.

### Laboratory tests

**Fig 3:**
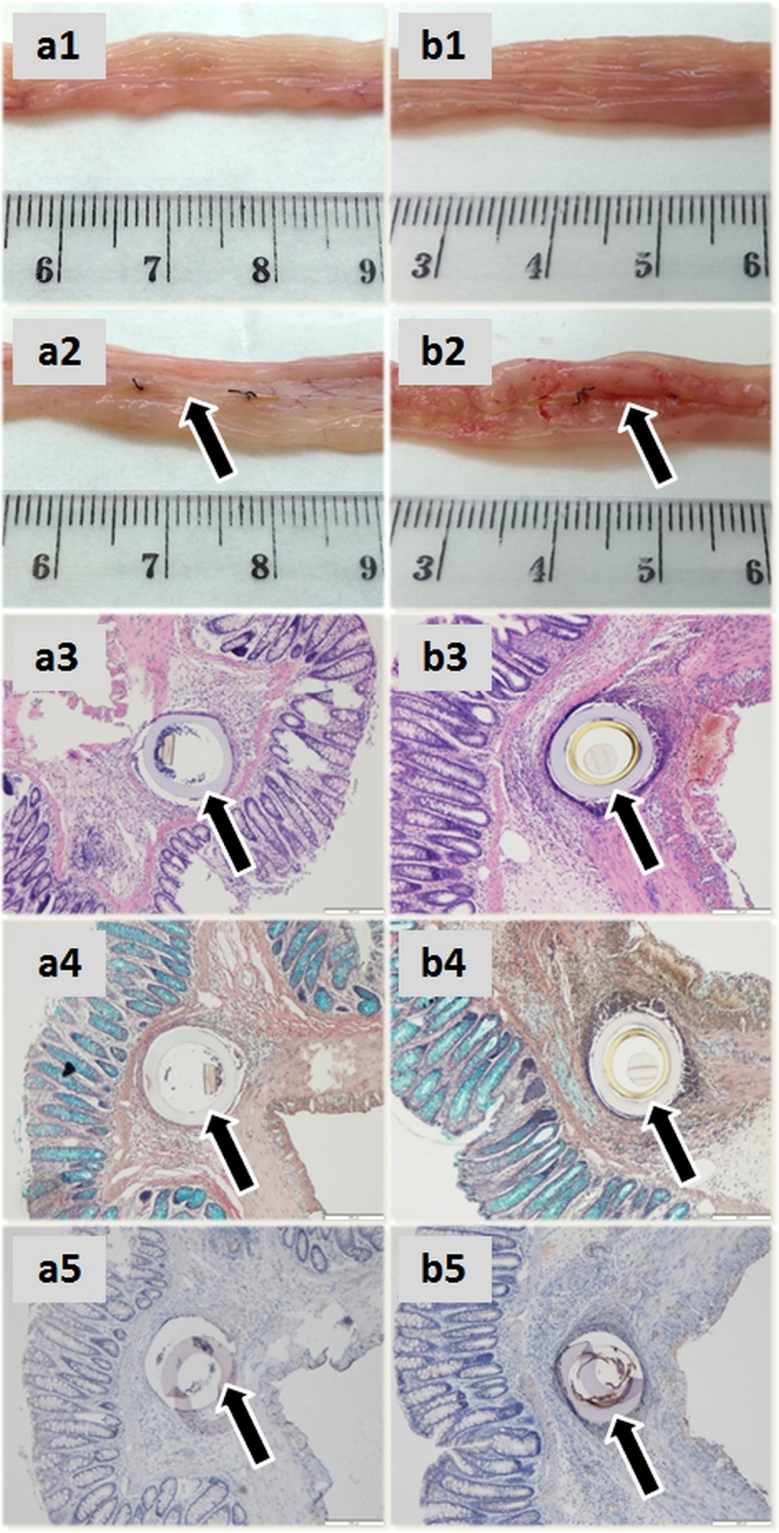
Morphological studies. Panels a1-5 illustrate macroscopic (mucosal, serosal) and microscopic (HE, Alcian Blue and iNOS) views of probe implantation site in the descending colon, respectively, of rat A, whereas b1-5 depict rat B2 tissues. The pictures show mixed (polynuclear + lymphocyte) inflammatory infiltration and submucosal granuloma formation of comparable magnitude with preserved goblet cells (Alcian blue positivity) and negative (rat A) to mild (rat B2) iNOS reactivity. Due to the low number of subjects, possible effect of 5-ASA in perfusate B could not be determined. Arrows point to the microdialysis probes (a2, b2) or their cross-sections (a3-5, b3-5, tissue processing artifacts are observable in a5 and b5).

In the eligible animals total colon length oscillated around 20 cm (24 cm, 18 cm and 18 cm for rats A, B1 and B2, respectively). According to post-mortem photographs of the large intestines the probe was implanted 4.7 to 8.5 cm from anus, *i*.*e*. in the descending segment of the organ. Representative photographs (rat A and rat B2) with detailed mucosal as well as serosal macroscopic and microscopic views of probe implantation site (featuring standard hematoxylin-eosin and Alcian Blue histochemistry and iNOS immunohistochemistry) are provided in Fig 3. The pictures show mixed (polynuclear + lymphocyte) inflammatory infiltration and submucosal granuloma formation with preserved goblet cells (Alcian blue positivity) and negative (rat A) to mild (rat B2) iNOS reactivity. Rat B1 presented with foreign body tissue reaction of analogous magnitude.

The results of plasma biochemistry and routine hematology are summarized in Table 4. With the exception of platelets (rat A), the table shows generally comparable findings between the subjects, with values in most cases within normal range [27,28]. Some trends toward mild anemia and hyperglycemia could be noted.

**Table 4:** Routine biochemistry and hematology.

Parameter (unit)		Glucose (mmol/L)	Lactate (mmol/L)	Urea (mmol/L)		WBC (10^3^/mm^3^)	RBC (10^6^/mm^3^)	HGB (g/dL)	HCT (%)	PLT (10^3^/mm^3^)	MCV (μm^3^)	MCH (pg)	MCHC (g/dL)
**Reference range**	* **	2.6–11 5.1–9.2	no data 1.9–5.6	4.0–8.2 4.0–9.3	‡ ‡‡	3.0–15 2.0–8.3	5.0–12 7.3–9.7	11.1–18.0 13.7–17.6	36–52 40–53	140–600 638–1177	44–69 49–58	12.0–24.5 17.1–20.4	21.6–42.0 32.9–37.5
**A**		8.9	4.0	4.8		2.9	5.8	11.5	34	175	59	19.8	33.6
**B1**		11.5	3.8	5.2		2.1	6.3	12.1	36	432	58	19.2	33.2
**B2**		10	5.0	6.4		2.5	5.7	11.7	34	418	60	20.6	34.1

Routine plasma or whole blood parameters were evaluated on day 5. Due to low number of subjects statistical evaluation was not performed and data were expressed as individual measurements. Abbreviations: WBC–white blood cells, RBC–red blood cells, HGB–hemoglobin, HCT–hematocrit, PLT–platelets, MCV–mean (red blood) cell volume, MCH–mean (red blood) cell HGB and MCHC–mean (red blood) cell HGB concentration.

*mean ± 2SD.

**2.5–97.5th percentiles.

‡ manufacturer´s datasheet.

‡‡range, *i*.*e*. (minimum—maximum).

For references see the text.

## Discussion

### Awake animal model of colon submucosal microdialysis

This work was designed to address the ongoing experimental challenge of large bowel monitoring by evaluating an awake rodent model of gut submucosal microdialysis. Intramural implantation of a thin linear probe type combined with the external apparatus allowed the animal to move freely within individual metabolic cage. In principle, this setup mimicked standard brain studies in small animals and provided an alternative to keeping the rats restrained [15]. Perfusate A composition, the rate of medium flow and sampling pattern respected earlier recommendations [21,29] and took into account our current analytical requirements. The duration of the study and the collection of specimens with focus on day 4 followed from the optimum time window between post-operative animal recovery and spatial development of foreign body reaction as reported by Mou *et al*. [24] and Kitano *et al*. [17]. In an attempt to mitigate such tissue reaction, one group of animals was locally administered anti-inflammatory perfusion fluid (perfusate B) containing 5-ASA. Most slides showed extensive mixed inflammatory infiltrate (granuloma) in the submucosal region surrounding the probe accompanied by iNOS up-regulation in the adjacent tissues (Fig 3). Due to low number of eligible subjects at the end of the study we were unable to determine whether the addition of 5-ASA had a preventative role in the development of local granuloma formation.

The unusually high drop-out rate observed in this study contrasts with the established technique of cerebral microdialysis [30]. This difference is mostly attributable to the functional anatomy of the rodent gut requiring a unique implantation procedure and specific catheter quality in terms of its dimensions and flexibility. In the brain, robust metallic probes of concentric design are usually fixed (glued) to the skull, whereas in the intestines, the probe should be as thin and malleable as possible and must be fixed by sutures within a pre-formed tunnel [3,19,31]. Correct technique of strictly submucosal channel formation requires longitudinal dilatation of intestinal tissue that has, in advance, been devoid of feces. Excessive manipulations of this kind may have had a negative impact on gut motility in the recovery phase and may have contributed to impaired bowel movements observed in several subjects.

In the present study the intramural approach was pursued. Even though intraluminal position is also possible in the intestines [8,11], it is not practicable for several-day-long awake studies due to the risk of semipermeable membrane clogging. Intraperitoneal approach, on the other hand, can be used for longer periods of time [32,33], yet it probably reflects global gut perfusion [14] and integrity [33] rather than local mucosal metabolism and microcirculation we were interested in. Although correct position of the sensor was apparent during each surgery, at the completion of the experiment, frequent transmural probe dislocation could not be prevented even by fastening one of the sutures less tightly to enable limited longitudinal probe movement in the pre-formed tunnel. In the rodent colon, this fact should not be very surprising, as relatively high incidence (in 15% of cases) of technical problems with the catheters (such as damage to the membrane, dislocation or incorrect probe placement) were registered by other experienced microdialysis users in short-term studies even in larger and more robust porcine tissues [34].

### Microdialysis and other laboratory data

Selected conventional analytes (glucose, lactate and urea) were employed to assess local microcirculation and provide some insight into probe function [8,20,21], although dynamic changes of submucosal metabolite recovery as long as four days following surgical probe implantation have not been described in rodents thus far. The present study shows that glucose and lactate-to-glucose ratio generally follow opposing trends with the former decreasing and the latter growing as soon as two to three days after surgery (Fig 2). Steady glucose decline is compatible with diminished microcirculation [20,34–37] or with progressive probe function loss, *i*.*e*. a drop in relative recovery of extracellular solutes [24], or with a combination thereof. The corresponding augmentation of lactate and lactate-to-glucose ratio supports the notion of impaired blood perfusion with spatially restricted anaerobic glycolysis [20]. For comparison, values of lactate-to-glucose ratio in ischemic rat stomach may come close to 1.5 [20].

Urea clearance has been advocated by Farnebo *et al*. as a convenient measure of local blood flow in a number of organs including the muscle, the skin or the liver [21,27,38]. Even though these authors found significant correlation between urea and lactate in the liver [21], the changes in the levels of urea during blood flow reduction were much less prominent (a typical increment of 10 to 30%) compared to the classical metabolic parameters or their ratios [20]. In the present study, the absolute concentrations of urea as well as RD_urea_ are displayed in Fig 2D. RD_urea_ was calculated from the last 8 values of urea concentration obtained on day 4 to decrease the bias owing to the absence of continuous plasma urea monitoring. The results suggest somewhat higher levels of delivery (*i*.*e*. negative recovery) than those estimated from the *in vitro* studies (S5 Fig), apparently due to a higher rate of urea diffusion in the perfused submucosal tissue in comparison with unstirred medium in the beaker. On day 4, relatively stable urea levels contrasted with steeper slopes of lactate-to-glucose ratio. Moreover, RD_urea_, and lactate-to-glucose ratio gave divergent results concerning local blood flow, albeit the differences were marginal. This discrepancy is in agreement with the lack of significant correlation between urea or RD_urea_ and glucose or lactate-to-glucose ratio in the three rats (Table 2). In order to verify the relationship between these parameters, more rats would have to be included in the study.

In the three eligible animals, macroscopic findings were satisfactory—total colon length was as expected, local alterations (gut wall thickness or organ deformities) were minimal and the intestinal tissues adjacent to the artificial tunnel with implanted catheter remained fully intact from mucosal as well as from serosal aspect (S4 Fig). Histology confirmed typical foreign body granulomas (Fig 3). Nonetheless, the technique, in general, presented with low reproducibility leading to a very small number of scientifically utilizable subjects. Statistical approach was, therefore, very limited and no firm conclusion regarding anti-inflammatory effect of locally administered 5-aminosalicylic acid could be drawn.

The results of plasma biochemistry and routine hematology were generally comparable (Table 4). Because of the lack of clinical platelet disorder signs and *in vitro* application of a typical anticoagulant, rather low platelet number in rat A may probably be explained by pseudothrombocytopenia [39], although microscopic analysis of the sample was not performed to confirm the diagnosis. Trends toward mild hyperglycemia and anemia may be attributable to operation stress.

## Conclusions

Microdialysis of rat gut is associated with considerable technical challenges that may lead to the loss of probe function and high drop-out rate. In this setting, limited data did not allow to draw any strict conclusion regarding local anti-inflammatory effectiveness of 5-ASA perfusion. Although intestinal microdialysis may be suitable for larger anesthetized animals, low reproducibility of the presented method compromises its routine experimental use in awake and freely moving small-sized rodents.

## Supporting information

S1 Fig Initial stage of the operation. The probe implantation technique involved: a, fixation of the Dacron® mesh button by sutures (arrow points to the protective plastic sleeve), b, tunneling of 27G needle in the submucosa of colon (the probe´s fiber skeleton enters the needle from its tip), c, positioning of the probe in the preformed tunnel (black arrow points to the semipermeable part of the probe, viewed also in detail in the inset with the grey arrow, white arrow shows the abdominal end of the protective sleeve) and d, fixation of the probe in place with atraumatic sutures.
(TIF)

S2 Fig Closing stage of the operation. The surgical procedure was finished by: a, placing the tubes of the probe into the protective plastic sleeve (arrow points to the strengthened and widened end of the tubing), b, suturing the abdominal opening, c, connecting the tubes (black arrow points to the free tubing of the probe, white arrow shows the applied silicone connector) and d, preparing the animal for transport into the metabolic cage (the arrow shows the protective metallic string).
(TIF)

S3 Fig Monitoring in the metabolic cage. Panel a provides a schematic overview of the microdialysis setting in awake rats. Dual channel swivel is mounted on a counter balance arm enabling unrestrained movement of the animal. Arrows indicate fluid flow. Panel b depicts an animal with a spring tether fixed at the nape of the neck. Black arrow points to the connection between the metallic spring tether and the plastic button, white arrow shows the dual channel swivel. Note: instead of using standard feeder assembly the feed was placed inside the cage to prevent damage to the implanted system and facilitate the animal´s recovery.
(TIF)

S4 Fig Sacrifice and autopsy. The figure illustrates: a, overview of the abdomen with arrow pointing to the healed wound of the operation, b, the scar from the previous midline laparotomy (black arrow) and the inflow and outflow tubes of the implanted probe (white arrow), c, normal situation of intraperitoneal organs with the place of entry of the tubing from the subcutaneous tunnel into the peritoneal cavity (arrow) and d, normal appearance of the gut with intact serosal surface (arrow) above the microdialysis probe. Correct position of the catheter in the submucosal space was also verified macroscopically (from the mucosal side) as well as using histology.
(TIF)

S5 Fig *In vitro* probe calibration. No-net-flux method of microdialysis probe calibration was used to determine relative recovery of the catheter for urea over a range of urea perfusate concentrations (main graph) and perfusion rates (graph inset). Relative differences between estimated and true urea concentrations (*i*.*e*. 10 mmol/L) are provided in the brackets. Main graph data represent means of two measurements per probe and flow rate. *In vitro* probe calibration showed ~25% recovery for urea at 1 μL/min perfusion rate and 25°C.
(TIF)

S1 File Microdialysis data. (XLSX)
