# Supplementary figures and images for: Critical evaluation of colon submucosal microdialysis in awake, mobile rats
Source: PLoS One. 2018 Jan 11;13(1):e0191041. doi: 10.1371/journal.pone.0191041 (PMC5764360; doi:10.1371/journal.pone.0191041)

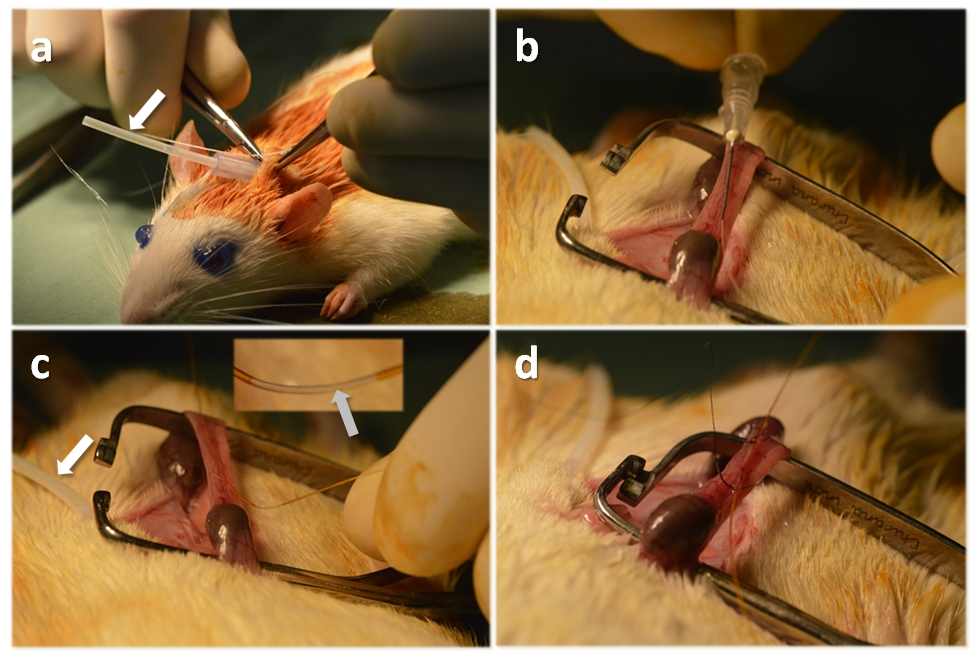

Supplement: S1 Fig — The probe implantation technique involved: a, fixation of the Dacron® mesh button by sutures (arrow points to the protective plastic sleeve), b, tunneling of 27G needle in the submucosa of colon (the probe´s fiber skeleton enters the needle from its tip), c, positioning of the probe in the preformed tunnel (black arrow points to the semipermeable part of the probe, viewed also in detail in the inset with the grey arrow, white arrow shows the abdominal end of the protective sleeve) and d, fixation of the probe in place with atraumatic sutures. (TIF) [file pone.0191041.s001.tif]

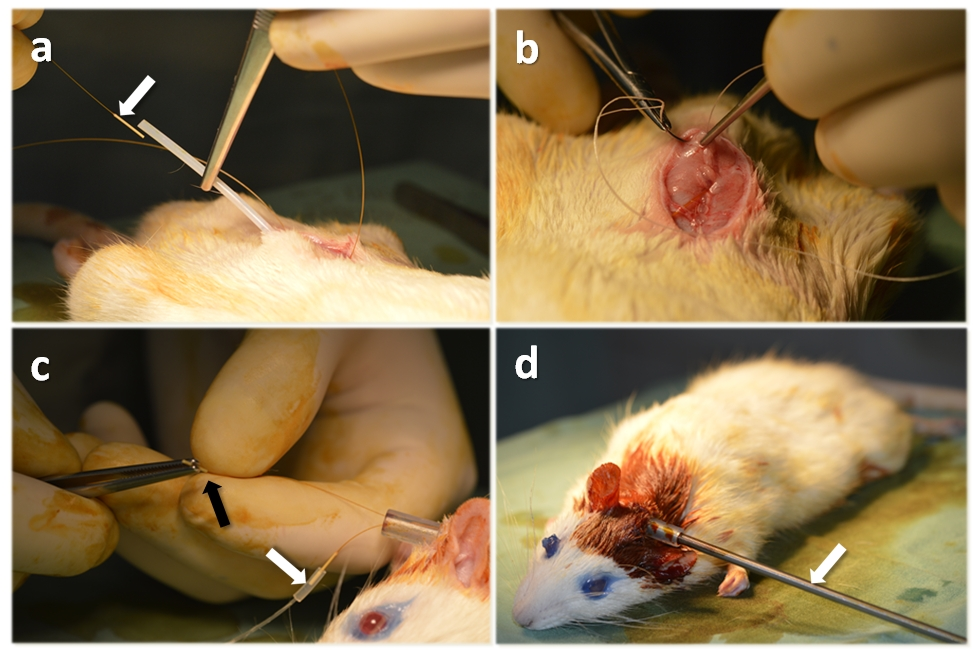

Supplement: S2 Fig — The surgical procedure was finished by: a, placing the tubes of the probe into the protective plastic sleeve (arrow points to the strengthened and widened end of the tubing), b, suturing the abdominal opening, c, connecting the tubes (black arrow points to the free tubing of the probe, white arrow shows the applied silicone connector) and d, preparing the animal for transport into the metabolic cage (the arrow shows the protective metallic string). (TIF) [file pone.0191041.s002.tif]

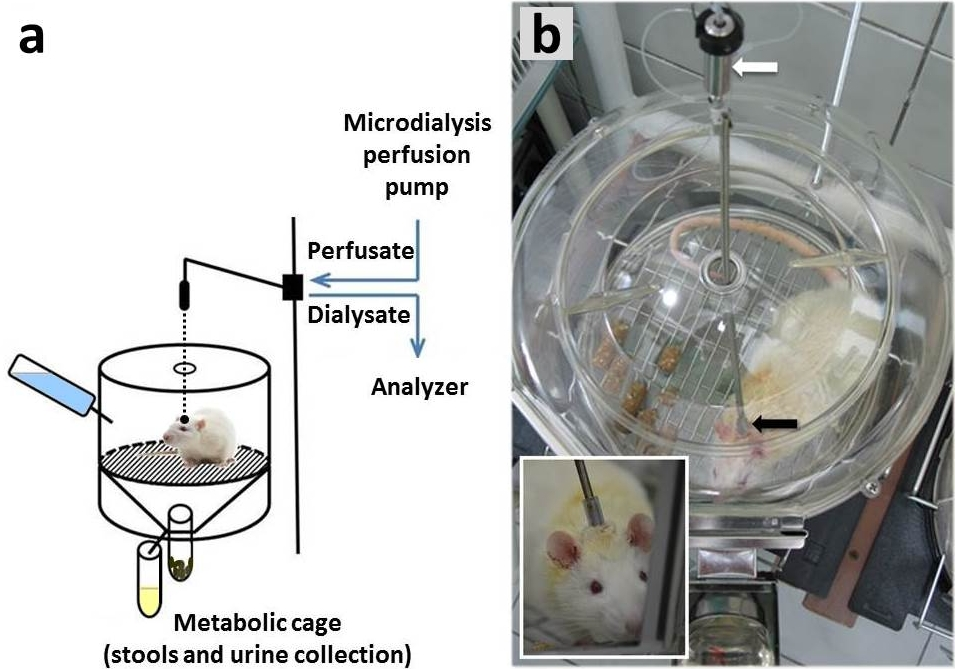

Supplement: S3 Fig — Panel a provides a schematic overview of the microdialysis setting in awake rats. Dual channel swivel is mounted on a counter balance arm enabling unrestrained movement of the animal. Arrows indicate fluid flow. Panel b depicts an animal with a spring tether fixed at the nape of the neck. Black arrow points to the connection between the metallic spring tether and the plastic button, white arrow shows the dual channel swivel. Note: instead of using standard feeder assembly the feed was placed inside the cage to prevent damage to the implanted system and facilitate the animal´s recovery. (TIF) [file pone.0191041.s003.tif]

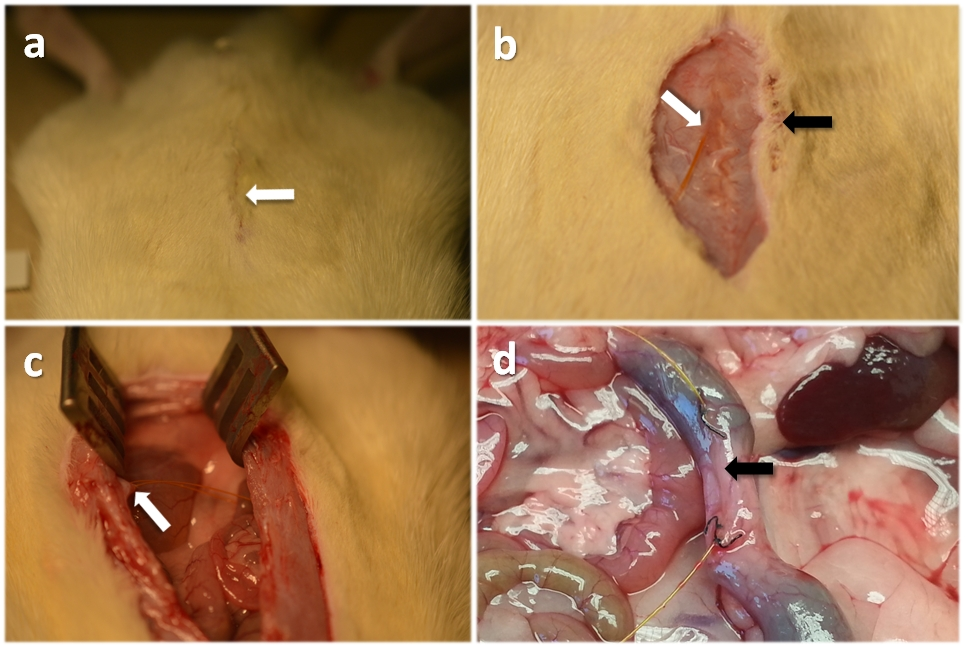

Supplement: S4 Fig — The figure illustrates: a, overview of the abdomen with arrow pointing to the healed wound of the operation, b, the scar from the previous midline laparotomy (black arrow) and the inflow and outflow tubes of the implanted probe (white arrow), c, normal situation of intraperitoneal organs with the place of entry of the tubing from the subcutaneous tunnel into the peritoneal cavity (arrow) and d, normal appearance of the gut with intact serosal surface (arrow) above the microdialysis probe. Correct position of the catheter in the submucosal space was also verified macroscopically (from the mucosal side) as well as using histology. (TIF) [file pone.0191041.s004.tif]
